# Crown and Bridge Work

**Published:** 1889-06

**Authors:** J. Marion Edmunds


ARTICLE III.

### CROWN AND BRIDGE WORK.

BY J. MARION EDMUNDS.

Crown and bridge work has long been a subject, not only interesting to the leaders of our profession, but has excited the attention, time and again, of the entire dental world. There have been hundreds of ingenious and practical devices in the construction of artificial crowns, as well as in the more advanced method of constructing and inserting artificial teeth, without plates—the so called bridge work. With these ingenious and improved ideas there has also kept pace corresponding development of manipulative skill, improvements in mechanical devices, and comprehensive and effective therapeutical agents. The recent consideration of our advanced thinkers are now bringing within the range of possibilities the practical application of this most valuable method of replacement. The cases suitable for the inserting of artificial crowns and bridge teeth, must be selected with caution and scientific skill. There are many roots that cannot be utilized as a means of support. The operation, therefore, is only of value where the root can be crowned, made perfectly inoffensive, comfortable, serviceable and durable to the patient. A pulpless root that is greatly denuded by absorption of the surrounding alveolus, much softened or very loose from extended destructive inflammation of the investing membranes, cannot be considered adequate to meet these requirements. This condition on the one hand, while on the other is that connected with impaired crowns, only partially broken down, with the pulp intact, the investing membrane free from disease, the cervical portion of alveolus and cementum unimpaired by decay, and firmly attached to the socket. Between these two extremes are gradations of normal and abnormal stages which may be operated upon, but the ultimate success of the operation will be more or less impaired, according to the condition of the root, and surrounding parts at the time of operating.

Therefore all prognostications should be based on a careful examination of the roots and surrounding parts. Any deviation from this method, excluding a recognition of these facts is delusive and unreliable. The results will not only be governed by the immediate condition of the root, but also by the general normal condition of the mouth. Any abnormal state of the hard or soft tissues, either by the presence of tartar, or any foreign deposit, will act as a predisposing cause in the development of unfavorable conditions. Whenever a root that has previously been treated for a chronic abscess, is selected to be operated upon, it is conservative treatment to open the pulp canal, completely fill it with cotton, the first piece having been saturated with a solution of chloride of zinc. This plugging of cotton should remain four or five days before crowning the root. If there is any inflammation of the gums around the teeth remaining, or if any are carious, appropriate treatment should be at once directed to them. Where a root or tooth has been restored to a normal condition, or where an inflammatory diathesis exists, the greatest caution should be used to avoid all unnecessary irritation. Irritation may be excited by files, saws, tapping of the mallet, slipping of corundum disks, the use of excising forceps, scalers, trimmers, and most surely by cutting the root below the margin of the gum. If loosening or tenderness of the root should take place, before setting the crown, it is best to defer the operation until all irritation or inflammation has subsided.

Suppuration is liable to follow irritation, then the removal of the crown becomes necessary, followed by a course of treatment and much annoyance to the patient, frequently involving the loss of the patient, through lack of confidence in both the operation and operator. The success of an operation may be greatly impaired by hurried, awkward, careless or injudicious manipulation.

Thus in cases where the remaining portion of a crown is improperly dressed with either a corundum disk, or any other instruments; or where the portion of a crown is left projecting, and the cap is made to fit it, in its ill shape. It will then project over and encroach upon the festoon of the gum, becoming a prime factor in setting up a dangerous inflammation. If in this case the crown had been properly dressed, slightly conical in shape, from the neck of the tooth to the articulating surface, and a closely fitting cap made to fit accurately around the neck of the tooth, the result would be perfect. While in the former case the result would be a miserable failure; and the opponents of crown and bridge work would quote this as valuable evidence of the worthlessness of the process, instead of awkwardness and ignorance.

Another important point to be considered is occlusion. If a crown is improperly set, there will be an injurious strain upon it. This may be by lateral pressure or direct antagonism, sometimes both, resulting in destructive inflammation, great distress to the patient, and the ultimate loss of the tooth.

Patients are not competent to judge either of the occlusion or adaptability of a crown in any case. It is therefore, the duty of the operator to note the proper articulation of the teeth and bear in mind the same for future reference. A patient's account of the condition is never to be relied upon; careful inspection only is to be depended upon. I have frequently had patients tell me a crown was "perfectly lovely" and "felt very comfortable," when it was an eighth of an inch too long, and the teeth, on the opposite side would not articulate by half an inch. If a living pulp remains in the root, the best and surest mode of operating is to cut, with a small corundum disk, a niche on the labial and palital surfaces, near the margin of the gum. Then at once nip the crown or portion of crown off, with the excising forceps. The operation of nipping the crown in this way is generally painless; when pain is felt, it is very slight.

The next important step is the removal of the pulp, which is easily accomplished without pain if care is taken. My method by which I have removed over two hundred living pulps is as follows:—I apply to the wounded pulp a solution of chloroform and cocaine, five grains of hydrochlorate of cocaine, to one dram of chloroform.

This solution must be applied directly to the bleeding pulp and renewed as often as necessary until the pulp is insensible to the point of the hypodermic needle. The needle may then be inserted into the pulp canal and three minims of ten per cent. solution of hydrochlorate of cocaine injected. From three to five minutes should be allowed for the action of the cocaine before extirpating the pulp. As soon as the bleeding has ceased, the canal may be enlarged and sealed with liquid gutta-percha and a few fibers of cotton. There are many methods of devitalizing a tooth or root. One of the most common is the application of arsenic. This should never be used, as it not only destroys the pulp, but the pericementum and all other vital connection of the root.

Another and more strictly barbarous method, is to *drive* with *one* blow, into the canal, a piece of orange wood, which has been previously shaped and dipped in creosote or carbolic acid. I would, however, advise all those, who are not in constant physical training, never to perform this operation on a *male* patient, who is in size and strength their superior. After the root has been prepared, the apical foramen sealed the canal enlarged to receive the dowel pin, and the crown selected, the next important step is fitting the crown. The method I consider best, is original with me, based on considerable experience and very carefully tested.

I have taken pleasure on different occasions to show several members of this Society some interesting cases, and shall try in the future as opportunities may present to continue to do so. The cervical portion of the root must be at least one sixteenth of an inch above the free margin of the gum to permit of proper shaping. The labial surface must first be ground to a bevel or semi-wedge shape, from the pulp canal to the margin of the gum.

The dowel pin is then inserted and the crown or facing, ground to fit the ground surface of the root. The facing must then be backed with platinum foil and fastened to the dowel pin with hard wax. The formula is—(white wax 8 oz., gum dammar 4 oz., resin 1 dram., melt the wax, then add the dammar, stir occasionally until all the dammar is dissolved, then add the resin.) A half ferule of 22 K. plate is then made to fit the palatal surface of the root closely. This may also be attached with hard wax to the dowel pin. The entire structure can now be removed and is ready to imbed, in plaster of paris and marble dust, pulverized pumice stone, sand or asbestos, one part of plaster to two of either of the latter ingredients. There should be no sharp corners, or square edges on the half ferule; they must be removed forming a neat curve from the centre of the summit on the palatal surface, to the base at the point of union, on either side, with the porcelain face. The square edges must be beveled very thin, and then burnished that no point of irritation remains. Neither must the ferule be inserted far beneath the free margin of the gum, or destructive inflammation of the periosteum will certainly ensue. It is necessary to try the crown, after it is finished, several times, pressing it well up to its place, noting every change in the color of the tissue, and removing any encroaching materials with the file; then again polishing and burnishing it before inserting. It is always best to have the ferule extend as little as possible under the gum, and the part that does so extend should be thin and fit very closely to the root.

Before leaving this part of my subject let me urge all those desiring to gain a reputation, not to adopt any of the cheap methods that are constantly advertised in the Dental Journals.

First, because a crown that has been properly selected in size and color, to correspond with the natural teeth on either side accurately, adapted to the absolute exclusion of all alimentary substances, is an invaluable operation to both patient and operator; and cannot be accomplished without skillful manipulation, the best and most desirable material, which all cheap articles tend to destroy.

Secondly, because cheaply constructed crowns that are made to fit by chance, take away from the operator his individuality and originality, therefore menace his manipulative ability and render him dependent upon the manufacturer, and unfit, when called upon to give the peculiar manipulative skill requisite of the practical and progressive dentist.

Bridge work is the substitution of artificial crowns for carious roots or teeth which have been extracted, a process by which the intervening spaces are filled in with a number of artificial crowns joined together, and permanently fixed in the mouth by attachment to one or more of the natural teeth or roots.

Among the Dental profession there is a great diversity of opinion in regard to the advisability of fixing such appliances permanently in the mouth. Some claim that it is uncleanly, contending that it is impossible to remove the constant accumulating oral debris, which must not only become offensive, but a source of injury to the remaining teeth. Resulting perhaps in their ultimate destruction by fermentation, putrefaction, decomposition and irritation. On the other side there are equally intelligent, competent, conscientious, and experienced dentists, who by long years of observation are thoroughly qualified and thereby entitled to their opinions. They meet these objections with positive denial of their existence, and commend in unqualified terms its excellence and superiority over all other methods of replacement when skillfully done under conditions favoring its insertion.

Among the admirers of this beautiful process I take the liberty to mention Prof. Joseph Richardson, M. D., D. D. S., Dr. Brophy, Dean of Chicago Dental College, Bing of Paris, France; Drs. Evans, Low, Starr, Dwinelle, Dexter, and Kingsley of this city, and many others.

The first account of bridge work is found in a report in the *Dental Cosmos* for Oct., 1869. This article stated that Dr. B. J. Bing, of Paris, had inserted a number of crowns in this manner, which had at that time been in the mouth nearly one year. Dr. Bing's method was to back a porcelain facing with 18 K. gold plate, soldering to the tooth to be inserted gold wire, either end resting in a cavity caused by decay or artificially formed for the purpose, the ends of which he builds into the cavities with gold foil.

The first record we have of bridge work in this country, is an operation by the late Marshall H. Webb, on Feb. 12th, 1873. This was a great improvement over Dr. Bing's method. This consisted of a porcelain facing backed with 18 K gold, a part of which projected on either side. To these projections he soldered gold wire, something in the form of the letter U. The pulps of the adjoining teeth were extirpated and these projecting wires inserted into the pulp canal. Then a small piece of gold plate, about the size of the neck of the root extracted, was made to fit the gum, then soldered with a mass of solder forming the palatal surface of the crown.

In the year 1886, thirteen years later, this crown was as firm as when inserted. Bridge work proper is only comprehensive manipulative skill, of a higher order, applied to the fundamental principles advanced by Drs. Bing and Webb,—improved upon, step by step, as those in the grand march of the dental profession recognized the importance of their calling, to supply the vast demand for more modern improvements, and conservative practice in the dental profession than had heretofore existed. Hundreds of the brightest minds that had previously been in a semi-torpid state responded to the call of advancement, each finding room in the vastness of the new field that was now opened for a multiplicity of ingenious devices in the construction and modes of attachment of artifical teeth to the remaining roots or teeth—thereby saving thousands of *useful teeth* that had previously been sacrificed to the forceps.

Bridge work is of two kinds, movable and immovable. Both have excellent points, the movable to my mind having somewhat the advantage in point of cleanliness, although there is no good reason why, if due care is observed, that the permanently fixed work cannot be kept as clean as the natural teeth. We all know by experience that the mouth is the most unclean part of the body even with those who pretend to take proper care of the teeth, and as to those who neglect this duty, the condition is simply one of disgust and filth And I think those who have practiced much in the Infirmary will agree with me, that language too strong can scarcely be used. Knowing this to be true, after the operator has put a mouth in fair order and charged his patient as to their responsibility in keeping the mouth clean, to my mind, he is in no way further responsible to the patient for any trouble that may be caused by negligence and uncleanliness. For if a patient is, by nature, neglectful and unclean about the mouth, the only one thing that will aid in reforming them, is frequent pressure on the pocket book, and with each assessment, a gentle remainder of the cause.

I will now try to describe my method of construction; a movable case of bridge work, the principles of which are entirely original with me. My first case was constructed in Sept., 1885.

The preparation of the teeth is most important. It is so in all crown and bridge work, particularly so in movable bridge work.

Special care must be taken that the tooth or root is slightly conical in shape from the neck to the grinding surface. Enough of the grinding surface is removed to permit the insertion of two caps. After the tooth has been shaped a strip of copper thirty-four U. S. standing gauge, a little wider than the tooth may be placed around the prepared tooth which may be tightly drawn into place with a pair of flat nose plyers. The copper is now removed and trimmed to the marks indicated by the plyers, which will give the requisite size of the gold plate to make the ferule or band. The next step is to force the ferule over the tooth, pressing and burnishing as necessary to properly adapt the ferule to the form of the tooth.

When the ferule has reached the gum the encroachment of its square edges will be indicated by a change of color in the tissue from pink to white. Now mark the ferule with an excavator around the festoon of the gum. The ferule must now be removed and trimmed to where it is marked. The edges should be filed and burnished before replacing it. This process must be continued until every part of the tooth is neatly covered, and slightly extending under the free margin of the gum. The edges must be beveled and burnished so there are no rough irritating points or edges remaining.

A neat impression may be taken by placing a small piece of wax on the grinding surface, pressing it firmly into place and allowing it to remain until cool. Then with a scaler or other suitable instrument remove the ferule and wax together. Run the ferule full of plaster and marble dust, cover it completely, except the wax which can be removed as soon as the plaster is set.

After the wax is removed, make a paper pattern, by pressing a piece of paper over the imbeded ferule, cutting it out as indicated by the impressed line. A piece of platinum foil is cut to this pattern and pressed into the open end of the ferule. which may have sufficient 18 K. gold solder flown over it to make a cap.

This completed, the cap is ready to be finished and cemented to the prepared tooth. Another cap is now to be made to telescope over the one in position. The ferule first being made, not quite extending to the gum. It must project at least one thirty-second of an inch above the surface. The wax may be used as I have previously directed, differing only in having the patient close their teeth upon the wax, thus securing an impression of the antagonizing molars or bicuspids. From this impression run a plaster model which can be duplicated in sand, and cast in zinc, making a die to strike up the cusps, for the movable or outside ferule. I find many advantages in making cusps in this way, from very thin 22 K. gold plate. After swaging the cusps and trimming them to fit the ferule, 20 K. solder may be flown in the depression of the under side making them solid. The cusps are then to be soldered to the ferule making a beautiful cap. One of the greatest advantages of this method of making cusps is that an operator can secure more perfect occlusion of the teeth that can be obtained in any other way. Because if the cusps are made from an impression of an antagonizing tooth, they are a fac-smile of the natural organs, and will perfectly articulate with the same. The details of this operation must be followed out with other teeth selected for attachment.

Supposing that two teeth, a right superior third molar, and a right superior first bicuspid, were capped and crowns made to telescope as above described, the crowns should be then placed over the caps, care being taken that proper occlusion had been obtained, an impression should be taken in the following manner:—mix the plaster to about the consistency of putty, pack this well around the crown and fill the spaces between them well. Now have the patient close the mouth, observing that the proper articulation has been secured. By these means the bite and impression is taken at the same time. After the plaster has set, request the patient to open the mouth which will generally break the impression. This is most always done. The pieces are then laid carefully aside until dry, when they may be put together with silex or wax at the option of the operator. The impression should now be varnished with shellac varnish, the formula is—(gum shellac 3 oz., alcohol one pint.) This makes a thick varnish, which is well adapted to this work. As the impression is now put together with the gold crowns imbedded in it and varnished, the next step is to place it in the articular, mixing the plaster medium and filling both sides at once, which makes an excellent model to work on. Next grind and fit the selected crowns to the model. Also grind the cusps off leaving a flat surface, and back them with platinum foil, allowing the foil to extend over the flat surface of the crown about one-eighth of an inch. Now wax these to the gold crowns with hard wax, and place the gold cusps, made in the manner described in proper position, over the porcelain facings, in such a manner as to secure perfect occlusion between the gold cusps and the cusps of the natural teeth. This being completed, the piece may be removed from the articulator, and imbedded in a matrix, made by mixing one part of plaster to two parts of marble dust. Then as soon as this sets, the wax can be removed and the matrix filled with 20 K. solder and covered with a piece of charcoal, that it may cool slowly.

After it is cooled it is ready for finishing and insertion. The advantages of this method will be readily appreciated by those who take into consideration the cleanliness and accessibility of the piece in case of accident when repair is needed. I have one more original device to describe in connection with movable bridge-work. I think this case is the largest on record.

Eight months ago, a lady consulted me regarding her mouth which was in a bad condition of ulceration, caused by a poorly constructed rubber plate which was neither useful nor ornamental.

There were only two superior third molars remaining in the arch; these were loose and the alveolus was in a state of suppuration. After due consideration I decided to try and save the teeth, and by my method replace the entire denture. After two weeks treatment I prepared the two teeth and capped them, after which I made gold crowns to telescope over the caps; I made a die and counterdie, swaged a rim 22 K. gold plate, 28 U. S. gauge in thickness by ⅜ of an inch in width to fit the arch. Placing this in the mouth and telescoping the crowns in their places, I secured a second impression and bite, which was placed in the articulator. After trimming and varnishing, the impression was removed by piece-meal, and countersunk teeth, waxed to the rim and crowns. These I removed from the articulator, placed in a flask and vulcanized in the usual manner, using pink rubber to attach the teeth to the gold rims and crowns. I have since that time treated the remaining two teeth, and I am happy to say with success, as they are becoming more firmly attached to their sockets. They are perfectly comfortable to the patient, suppuration having long since ceased, notwithstanding the strain of an entire denture upon them.—*N. Y. Dental College Record.*

